# The conceptual framework for the therapeutic approach used in phase 3 trials of MDMA-assisted therapy for PTSD

**DOI:** 10.3389/fpsyg.2024.1427531

**Published:** 2024-11-04

**Authors:** Kelley C. O’Donnell, Lauren Okano, Michael Alpert, Christopher R. Nicholas, Chantelle Thomas, Bruce Poulter, Ann Mithoefer, Michael Mithoefer, Marcela Ot’alora G

**Affiliations:** ^1^Department of Psychiatry, NYU School of Medicine, New York City, NY, United States; ^2^Department of Training and Supervision, Lykos Therapeutics, Inc., Santa Cruz, CA, United States; ^3^Department of Psychiatry, Beth Israel Deaconess Medical Center and Harvard Medical School, Boston, MA, United States; ^4^Department of Family Medicine and Community Health, University of Wisconsin School of Medicine and Public Health, Madison, WI, United States; ^5^Transdisciplinary Center for Research in Psychoactive Substances, University of Wisconsin, Madison, WI, United States; ^6^Aguazul- Bluewater, Inc., Boulder, CO, United States; ^7^Department of Psychiatry and Behavioral Services, Medical University of South Carolina, Charleston, SC, United States

**Keywords:** MDMA-assisted therapy, post-traumatic stress disorder, inner healing intelligence, inner-directed, non-pathologizing stance, midoafetamine, trauma-informed care, psychedelic-assisted psychotherapy

## Abstract

Results from multiple recent studies support further evaluation of 3,4-methylenedioxymethamphetamine (MDMA) in conjunction with psychotherapy (i.e., MDMA-Assisted Therapy) in the treatment of post-traumatic stress disorder (PTSD). In two Phase 3 trials, MDMA-Assisted Therapy comprised a short-term, intensive psychotherapy that included three sessions directly facilitated by MDMA (referred to as “experimental sessions”), as well as a number of non-drug psychotherapy sessions. This treatment model aimed to harness the potential of MDMA to facilitate recall and processing of traumatic memories, and to increase learning in a social context, integrating “top-down” and “bottom-up” approaches to trauma-focused care. To date, the conceptual framework for this treatment has not been described in the scientific literature. This omission has contributed to misunderstandings about both the theoretical underpinnings of this modality and the therapeutic approach that emerges from it. This paper delineates the psychotherapeutic concepts, theories, and historical antecedents underlying the inner-directed approach to MDMA-Assisted Therapy for PTSD. Broadly speaking, this therapeutic framework centered the concept of the participant’s inner healing intelligence as the primary agent of change, with the therapeutic relationship being the core facilitative condition fostering the participant’s self-directed movement toward recovery and growth. Corollaries to this holistic, self-directed, relational, and trauma-informed framework include a non-pathologizing approach to the participant’s embodied experience (including the possibility of intense emotional and somatic expression, experiences of multiplicity, suicidal ideation, and multigenerational and transpersonal experiences), as well as the therapists’ own psychodynamic, somatic, and transpersonal awareness, empathic attunement, relational skillfulness, and cultural humility. The use of MDMA in conjunction with this psychotherapy platform outperformed the use of placebo with psychotherapy in Phase 2 and 3 trials, as measured by symptom reduction in participants with PTSD. However, within-group comparisons also identified significant symptom reduction in participants who did not receive MDMA, lending empirical support to the psychotherapy model itself. In addition to comparative efficacy trials, future research should investigate which elements of the conceptual framework and therapeutic approach underlie the clinical benefit in individuals with PTSD.

## 1 Introduction

Helping, fixing, and serving represent three different ways of seeing life. When you help, you see life as weak. When you fix, you see life as broken. When you serve, you see life as whole. (Remen, 2021)

Results from multiple recent studies support further evaluation of 3,4-methylenedioxymethamphetamine (MDMA) in conjunction with psychotherapy—i.e., MDMA-Assisted Therapy (Box 1)—as a treatment patients with post-traumatic stress disorder (PTSD). In two Phase 3, double-blind, randomized controlled trials (RCTs; Mitchell et al., 2021; Mitchell et al., 2023; n = 90 and n = 104, respectively), participants who received MDMA in conjunction with an intensive, highly specialized psychotherapy (described below) were found to have significant attenuation of PTSD symptom severity relative to those assigned to the placebo + psychotherapy group. The between-group effect sizes were robust in both Phase 3 trials (*d* = 0.91 and *d* = 0.7, respectively), adding to similar findings from multiple Phase 2 studies of MDMA-Assisted Therapy for PTSD (Mithoefer et al., 2011; Mithoefer et al., 2018; Mithoefer et al., 2013; Ot’alora et al., 2018). Both treatment groups in the Phase 3 trials also had much lower dropout rates (1.9% [1/53] in the MDMA-Assisted Therapy group and 15.7% [8/51] in the placebo with psychotherapy group; Mitchell et al., 2021; Mitchell et al., 2023) than those typically observed in clinical trials of prolonged exposure therapy (55.8%) and cognitive processing therapy (46.6%; Schnurr et al., 2022), the gold standard treatments for patients with PTSD.

BOX 1 **What is MDMA-assisted therapy?**

Although MDMA is not a classic psychedelic (Nichols, 1986; Kirkpatrick et al., 2014) its clinical use in contemporary research falls under the rubric of psychedelic-assisted therapy, namely, “a clinical model of treatment that attends to set, setting, and dose in an effort to occasion a substance-induced non-ordinary state of consciousness (NOSC), embedded within a supportive framework that includes preparation for and integration of the NOSC” (O’Donnell et al., 2023). This basic framework for psychedelic treatment was used in early reports of clinical MDMA use, prior to its scheduling by the Drug Enforcement Administration (Greer and Tolbert, 1998; Passie, 2018). It underwent significant expansion and development before, during, and after the Phase 2 trials of MDMA-Assisted Therapy for PTSD. In Phase 2 and Phase 3 trials, the treatment comprised 2–3 8-h “experimental sessions” that were explicitly facilitated by MDMA (vs. placebo + psychotherapy), as well as multiple 90-min non-drug psychotherapy visits—referred to as “preparation” and “integration” therapy sessions—that took place before and after the experiential sessions, respectively. Facilitated by two trained therapists (Box 5), all therapy sessions also attended to various aspects of “set and setting” (Box 2), some of which are common to most psychedelic-assisted therapy models, and others of which were emphasized in a trauma-informed (Box 4) effort to meet the specific needs of this clinical population. That said, neither the classic “set and setting” (Box 2) model nor a straightforward “trauma-informed” approach captures the full conceptual framework of the therapeutic model used in the Phase 3 trials of MDMA-Assisted Therapy for PTSD, as further delineated in the main text.

In addition to the large effect sizes of MDMA-Assisted Therapy for moderate and severe PTSD, within-group analyses of the Phase 3 data indicated that participants in the placebo + psychotherapy arms of the two trials *also* exhibited a significant therapeutic benefit (*d* = 1.25 for placebo with psychotherapy vs. *d* = 1.95 for MDMA with therapy; Mitchell et al., 2021). Though lower than the effect size in the MDMA-Assisted Therapy groups, the clinical benefit conferred by psychotherapy alone was higher in the placebo with psychotherapy group (*d* = 1.25; Mitchell et al., 2021) than in prior evidence-based psychotherapy trials for PTSD (*d* = 0.17; Cavarra et al., 2022; Horton et al., 2021). This finding is particularly remarkable given these participants’ high-risk status (as measured by prior suicidality) and treatment history (as measured by the number of years in psychotherapy, and by the number of evidence-based interventions—pharmacotherapy and psychotherapy alike—reported before enrolling in the Phase 3 trials). These clinical outcomes and retention rates do not imply that the psychotherapy platform was superior to other treatments for PTSD; however, they do challenge the claim (McNamee et al., 2023) that this psychotherapy platform lacks an empirical basis, and suggest that further study of the therapeutic approach^1^ is warranted.

That claim has nevertheless been made—perhaps due to the paucity of published literature describing the therapeutic approach and its conceptual framework (Cavarra et al., 2022; Horton et al., 2021), as well as a frequent use of the term “nondirective” when describing a “set and setting” model of psychedelic-assisted therapy (Box 2; Horton et al., 2021; Deckel et al., 2024). Indeed, a colloquial reading of “nondirective” has little in common with its use in this intensive, highly specialized treatment. This paper describes and synthesizes the core concepts underlying the therapeutic approach to MDMA-Assisted Therapy in Phase 3 trials, identifying different schools of thought^2^ (Boxes 2,3) that contributed to its development. A subsequent paper will further describe the integrative therapeutic approach that emerged from this framework.

BOX 2 **Set, setting, and matrix.**

Psychedelic-assisted therapy studies to date have emphasized the importance of “set” (i.e., the participant’s internal state at the time of the session), and “setting” (i.e., the many extrinsic, non-pharmacological factors that influence a participant’s experience in a non-ordinary state of consciousness; O’Donnell et al., 2023). Of note, the phrase “set and setting” originally applied to the psychedelic experience itself (Hartogsohn, 2017); however, within the context of MDMA-Assisted Therapy, the concept is no less important for the non-drug psychotherapy sessions, consistent with the “common factors” model of psychotherapy delineated by Frank et al. (1993). Specifically, the environmental and interpersonal aspects of “set and setting” are represented in the “common factors” of a “healing context” and a functional (“working”) alliance, respectively. With respect to the interpersonal aspects, in Frank’s model the therapist takes on the role of a “healer” who provides a culturally acceptable explanation of the patient’s distress. Consistent with that model, and with the concept of relational ethics (Pollard, 2015), the therapists in the Phase 3 trials were thoughtful about the ways that therapeutic “priming” and efforts to acculturate the patient to certain ideas (e.g., the inner healing intelligence”) may shape and be shaped by the therapeutic framework and relationship, power dynamics, cultural diversity, therapeutic outcomes, and other factors that may be common to different psychotherapies (Wolff et al., 2024).Please see the main text, including Footnote #3, for further discussion of these topics.

As in prior psychedelic research, many other contextual factors were also considered in the Phase 3 trials of MDMA-Assisted Therapy. These included careful selection of the physical location of all therapy sessions (i.e., a private, comfortable, non-clinical setting); the use of two therapists (Box 5); the thoughtful, culturally sensitive incorporation of ritual/ceremony; and the use of music to dynamically amplify and support the MDMA-Assisted Therapy experience (Kaelen et al., 2018). In addition to their current and historical life circumstances, participants’ trauma history and current symptomatology, as well as their past and present intrapersonal (i.e., emotional, cognitive, somatic) experiences, and their interpersonal, transpersonal, and sociocultural experiences and relationships, were all among the crucial factors influencing their “set.” That “set” was in turn dynamically shaped by the content and process of each therapy session.

Adding to the original concept of “set and setting” in psychedelic therapy, pioneering psychedelic clinician-researcher Eisner (1997) introduced the concept of “matrix,” namely, “the environment from which the [participant] comes, such as family and living situation; the environment the [participant] is living in while having sessions; and the environment to which a [participant] returns after successful therapy” (p. 215). Though limited by the inherently individualistic framework of biomedical research, investigators on the Phase 3 trials were nevertheless mindful of the external “matrix” of sociocultural conditions that might facilitate and/or impede the participant’s recovery process within and outside the therapeutic milieu.

BOX 3 **Therapeutic “lineage.”**

The therapy model used in the Phase 3 trials was developed not through a straightforward synthesis of different bodies of semantic knowledge, but through the experience and practice of a small but diverse group of clinician-researchers (primarily authors AM, MM, MO, BP) who conducted the majority of the Phase 2 MDMA-Assisted Therapy sessions (Mithoefer et al., 2011; Mithoefer et al., 2018; Mithoefer et al., 2013; Ot’alora et al., 2018; Mithoefer et al., 2019; Oehen et al., 2013) that laid the groundwork for the Phase 3 trials. This core group of investigators—who went on to train, in the initial intensive training and/or the subsequent longitudinal process of clinical consultation, all of the clinician-investigators involved in the Phase 3 trials—brought to their practice of MDMA-Assisted Therapy their own prior experience with myriad healing modalities. These included, but were not limited to, psychodynamic, humanistic, interpersonal, experiential, and transpersonal psychotherapies (Bland and DeRobertis, 2020), particularly as synthesized in the psychedelic therapy of Grof (1975) and Naranjo (1974); holotropic breathwork (Grof and Grof, 2010); Gestalt Therapy (Perls et al., 1994); Hakomi Mindful Somatic Therapy (Kurtz, 2007); Internal Family Systems Therapy (Schwartz, 1995); process-oriented psychology (Mindell, 1988); Rolf Structural Integration (Rolf and Feitis, 1990); Trauma Energetics (Redpath, 1995); and a variety of Buddhist traditions (Suzuki, 2006; Chödrön, 2019; Nhất Hạnh, 1991).

Although MDMA has not historically been used by Indigenous communities, these investigators were nevertheless indirectly influenced by a variety of psychological frameworks and traditions situated outside the biomedical model (Redpath, 2019; Naranjo, 1974; Grof and Valier, 1984; Jung, 2019), many of which overlap conceptually with Indigenous healing traditions. For example, Stanislav Grof, Claudio Naranjo, and William Redpath, whose work directly influenced the conceptual framework for MDMA-Assisted Therapy in the Phase 2 and Phase 3 trials, drew from a wide range of psychological, cultural, and psychospiritual traditions—Eastern and Western, ancient and modern alike—as they developed and refined their own holistic interpretive lenses and therapeutic practices. In addition to the concept of the inner healing intelligence, their integrative frameworks contributed to the therapeutic approach in the Phase 3 trials in a number of ways, including: the understanding of therapeutic “progress” as a nonlinear phenomenon; the importance of a therapeutic “container” that fosters a sense of safety, curiosity, and possibility; the acknowledgement of the intangible (i.e., invisible, spiritual, mystical) dimensions of human experience and healing; and a permissive—even welcoming—attitude towards intense emotional and embodied expression (e.g., spontaneous movement). Many of these factors dovetail with Indigenous healing traditions. However, none of the core investigators were directly trained in any Indigenous or other shamanic lineage(s), and all were sensitive to the potential for (and dangers of) cultural appropriation within and outside psychedelic spaces.

In addition to, though no less important than, their own clinical training and experience, each investigator had also been shaped by their unique familial and cultural heritage (Catherall and Pinsof, 1987); their intrapersonal, relational, and sociocultural backgrounds; and their past and ongoing inner work, including personal psychotherapy and/or other experiential, contemplative, spiritual, and healing practices (Dimidjian and Linehan, 2003). All of these factors, as well as their individual and collective experiences working on earlier studies of MDMA-Assisted Therapy for PTSD, contributed to the conceptual framework described in this paper, and the therapeutic approach that will be described elsewhere.

Given the diversity of influences on these core investigators, no single therapeutic “lineage”—or any concrete genealogy—can accurately trace its development. Rather than a genetic metaphor, we might more appropriately consider the process by which individual bees draw nectar from different sources, storing and digesting it before sharing it with the hive, where others process it further. In this iterative process of digestion and generation, the bees collectively produce honey.

### 1.1 Key concepts

The treatment model used in the two Phase 3 trials of MDMA-Assisted Therapy for patients with PTSD has at its core a number of key therapeutic components. Though these can be described in different ways, they can generally be identified as follows: a non-pathologizing approach to the participant’s experience—including any manifestations of multiplicity (described below), transpersonal phenomena, and/or somatic expression; respect for the participant’s autonomy in the therapeutic process; and attention to the many intrapersonal and contextual factors that dynamically contribute to that process. These principles emerge out of two conceptual pillars of the framework, namely, the concept of the *inner healing intelligence* and *relationality*. In the sections below, we discuss each of these in turn. In addition, because the Phase 3 trials studied the effects of MDMA-Assisted Therapy in participants with PTSD, careful attention was also paid to the principles of trauma-informed care (Box 4).

BOX 4 **Trauma-informed care.**

For individuals with a history of trauma, the healthcare setting itself can be a source of distress and re-traumatization. Rooted in trauma theory, the framework of trauma-informed care prioritizes the survivor’s safety, trust, and empowerment in the service of healing (Herman, 2015; Catherall and Pinsof, 1987). It also explicitly recognizes the societal factors that generate and exacerbate traumatic experiences (Rosenthal et al., 2016), despite the fact that these factors cannot be addressed in individual psychotherapy.

Survivors of interpersonal trauma, including identity-based traumas, may struggle to experience safety and trust. The trauma-informed therapist cultivates both through their interpersonal style, cultural humility, communication skills, and emotional availability, as well as their ability to welcome and tolerate intense affects and trauma narratives. In trauma-informed care, therapists foster an egalitarian therapist-participant relationship, empowering the participant to determine the direction and pace of change (Center for Substance Abuse Treatment (US), 2014; Reeves, 2015). By various means—e.g., centering the participant’s autonomy and their active, ongoing consent; openly addressing potential power dynamics related to one’s own cultural identity and social location; using openness and judicious self-disclosure, as well as flexibility regarding the techniques and pace of treatment—therapists practicing MDMA-Assisted Therapy strive to build a balanced therapeutic relationship that fosters the participant’s felt sense of safety and empowerment. The two therapists support the participant’s self-directed healing process, modeling the very trust, openness, non-judgmental patience, and compassionate curiosity they are inviting the participant to explore and practice.

Within a trauma-informed framework, therapists are also aware that individuals with a history of complex and persistent trauma may struggle with self-esteem and/or self-trust, which can impact their feeling of being worthy of care. They may also struggle with feelings of aggression, which may be internalized, disowned, or directed towards the therapist(s; Herman, 2015; Doob, 1992). Any of these can affect the therapeutic alliance, particularly if the therapist is not able to tolerate the participant’s negative affect and/or their ambivalence regarding treatment (Kuchuck, 2021; Strupp, 1980). For these reasons, a therapist practicing MDMA-Assisted Therapy in this model cultivates self-awareness within and outside the session, bringing curiosity to their own internal experience in the treatment, and seeking outside help as needed to ensure such experiences might serve, rather than undermine, their efforts to nurture the therapeutic relationship.

## 2 The concept of inner healing intelligence

### 2.1 Organicity

The term “inner healing intelligence,” first used by the Czech psychiatrist, psychoanalyst, and psychedelic pioneer Stanislav Grof, who himself credited Carl Jung for the concept (Grof, 2007), stems from a core axiom, namely, that each person has an innate tendency toward self-directed healing and growth. As used in this theoretical framework and in this paper, the term “inner healing intelligence” refers to that tendency and process. The concept is central to all humanistic, experiential, and transpersonal psychotherapies, which in turn build on and integrate various ontologies, epistemologies, and practices—including Eastern, Western esoteric, and Indigenous traditions (Box 3) that long predate modern psychotherapy and the MDMA-Assisted Therapy model described here. The core principle from which the concept of the inner healing intelligence emerges has been called *organicity*, a term coined by the physician Kurt Goldstein in his book “The Organism” (Goldstein and Goldstein, 2000). In that seminal text, Goldstein described the body’s remarkable capacity to adapt physically and psychologically in response to psychological and neurological trauma. He attributed that capacity to organicity, a principle that came to underlie many contemporary schools of psychotherapy and form the basis of psychotherapy process research (Perls et al., 1994; Maslow, 1968; Weiss et al., 2015; Rogers, 1965).^3^ The term refers to the fact that human beings are living *organisms*, and as such, they are possessed of certain properties. Specifically, each person is: (1) fundamentally whole, yet (2) composed of individual, interdependent parts; they also (3) possess an innate propensity to grow and flourish in (4) a dynamic relationship with their changing environment.

Central to an organismic framework—and to the therapeutic orientation for the Phase 3 trials—is this concept of an individual as a self-organizing, self-directing, and self-equilibrating system that is fundamentally whole (Maslow, 1968; Kurtz, 2007). Within this framework, inner healing^4^ intelligence refers to the process^5^ by which that whole internal system is dynamically maintained within—and is in constant communication with—larger systems. This concept is also elaborated in other psychological frameworks (e.g., in Carl Jung’s “transcendent function”; Jung et al., 1953), Abraham Maslow’s discussion of the human capacity for self-actualization and self-transcendence (Maslow, 1968), and a number of other holistic approaches drawing from both Eastern and Western healing traditions (Weiss et al., 2015; Redpath, 2019; Levine, 1997; Kuriyama, 2011; Mindell, 2011).^6^

#### 2.1.1 Organicity and the non-directive approach

Within a framework of organicity, humanistic psychologist Carl Rogers proposed that a therapist’s^7^ approach should be “nondirective,”^8^ meaning that their role is not to force or *direct* change, but to provide the “facilitative conditions” required to support an individual’s natural, *self*-directed process of organismic healing and growth (Rogers, 1970). Within such a framework, the therapist therefore has an *active* role—for example, in generating a sense of safety in the therapeutic milieu (Center for Substance Abuse Treatment (US), 2014; Herman, 2015), or leading a breathing exercise at the participant’s request—but not a *directive* role, because the content of the participant’s unfolding experience is *directed* by their own inner healing intelligence. For this reason, the therapeutic process is described as “*inner*-*directed*” (or “*self*-*directed*”). Though a full description of the therapist’s role in an inner-directed approach is outside the scope of this paper, certain aspects of it are discussed in the sections below.

### 2.2 Non-pathologizing stance

Conventional models of psychological suffering often pathologize a patient’s experience, conceptualizing them as “treatment-resistant,” or implicitly “broken”—and therefore labeled as having “failed” prior treatments and as being in need of “fixing” by the therapist or another external agent (Remen, 2021; Rosenthal et al., 2016). Such a stance amplifies the power dynamic that is already inherent to the therapeutic relationship, risks causing harm (Pollard, 2015), and is antithetical to the values represented in this theoretical framework. By contrast, within an organismic framework that sees each participant as fundamentally whole, a non-pathologizing stance is one of the facilitative conditions that the therapists bring to bear in the service of a participant’s own, inner-directed trauma processing and growth. Such a stance supports the full expression of a participant’s experience(s) in MDMA-Assisted Therapy, including potential experiences of multiplicity, transpersonal phenomena, intense emotional and somatic expression, and complex relational dynamics.

#### 2.2.1 Multiplicity and the non-pathologizing stance

The concept of multiplicity is another core feature of the conceptual framework for the therapeutic approach conducted in the Phase 3 trials. “Multiplicity” refers to a psychological phenomenon that has been parsed in various ways by different theoretical schools—including the “subpersonalities” and the “ego-” and “self-states” described in psychoanalytic, humanistic, transpersonal psychology (Grof, 1980; Kuchuck, 2021; Rowan, 1990; Bromberg, 1996; Mitchell and Black, 1995; Stern, 2017), the psychosynthesis of Assagioli (1973), and the Gestalt Therapy and related work of Perls et al. (1994), Naranjo (1974), Naranjo and Mollart Rogerson (2020) and others; the “complexes” and “archetypes” of the analytical of Jung’s (1969) and archetypal of Hillman (1992) psychologies; as well as the “modes” in Schema therapy (Edwards and Arntz, 2012), the “selves” in Voice Dialogue (Stone and Stone, 1998), and the “parts” in Internal Family Systems Therapy (Schwartz, 1995).^9^ The concept of multiplicity should not be equated with the constellation of symptoms constituting the DSM-5 diagnosis of “dissociative identity disorder” [American Psychiatric Association, 2013; formerly “multiple personality disorder” (American Psychiatric Association, 1998)]. On the contrary: multiplicity *per se* is a normal phenomenon, and as such, therapists working on the Phase 3 trials did not pathologize any expressions of it. This non-pathologizing stance toward multiplicity was particularly important because the experience is frequently amplified for individuals with PTSD (Herman, 2015; Bromberg, 1996; Stern, 2017; Schwartz, 1995), and may be transiently intensified in the presence of MDMA (Mithoefer, 2013).

#### 2.2.2 Transpersonal experiences and the non-pathologizing stance

Though the phenomenology of an MDMA experience differs from that of classic psychedelics, they share many features (Studerus et al., 2010). These include the potential for transpersonal experiences—i.e., experiences, often felt to be “spiritual” or transcendental in nature, in which a person’s felt experience extends beyond the ordinary bounds of their individuality, and/or beyond the ordinary limitations of time and space (Grof, 1975). Across cultures and eras, such experiences—whether spontaneous or occasioned by psychedelic substances or other means (e.g., contemplative practices, religious and/or communal healing ceremonies, breathwork, shamanic drumming, sweat lodges, extreme fasting, etc.)—have been sought and harnessed in the service of somatic and psychospiritual healing and change (James, 2003; Hastings, 2003; Muraresku, 2020). However, such experiences have no clear role in a reductionist behavioral or classic Freudian psychoanalytic framework.^10^ As such, clinicians trained exclusively in these schools may be unfamiliar with the possibility and phenomenology of transpersonal experiences, particularly in a psychotherapy setting, and may therefore be inclined to pathologize such experiences if they emerge in an MDMA-Assisted Therapy session. However, given the frequency with which they do emerge (Mithoefer, 2013), coupled with their potential therapeutic value (Ross et al., 2016), the model in the Phase 3 trials extended the non-pathologizing approach to transpersonal experiences. That said, unlike other, explicitly spiritual uses of MDMA and other psychedelics in some Western (Muraresku, 2020; Adamson and Metzner, 1988; Metzner et al., 1999) and Indigenous (Labate, 2018; Feinberg et al., 2018; Halifax, 1979; Tupper, 2002) contexts, the therapeutic framework for the Phase 3 trials conceptualized MDMA—used in conjunction with psychotherapy—not as a sacrament, but as a catalyst for processing traumatic memories and experiences in the setting of the therapeutic relationship. As such, the therapeutic approach to MDMA-Assisted Therapy did not deliberately occasion transpersonal experiences. Instead, drawing from the concept of the participant’s inner healing intelligence, and consistent with various experiential schools of psychotherapy (Perls et al., 1994; Weiss et al., 2015; Levine, 1997; Gendlin, 2007), this model emphasized the importance of bringing non-judgmental curiosity and “beginner’s mind” (Suzuki, 2006; Linehan, 2015) to the participant’s moment-to-moment experience, rather than seeking to guide it toward one or another “type” of experience.

### 2.3 Organicity and the fullness of experience

The organismic orientation, like any interpretive and therapeutic framework, represents a certain set of values, and with them an ethical stance taken by the therapists in support of the participant (Pollard, 2015). This framework assumes, welcomes, and attends to all aspects—cognitive, emotional, relational, and transpersonal, and embodied (i.e., interoceptive and somatic)—of the participant’s experience as it unfolds in the treatment setting. Any of these aspects may be affected by trauma (Redpath, 2019; Center for Substance Abuse Treatment (US), 2014; Herman, 2015) and different trauma-based treatments—e.g., cognitive, or “top-down,” and somatic, or “bottom-up,” approaches—aim to restore equilibrium among them. Within the organismic framework of the Phase 3 trials, therapists drew from complementary orientations in the service of self-directed change. For example, in a number of evidence-based approaches to PTSD treatment, imaginal exposure to, and explicit recall of, traumatic experiences are both thought to be important for the trauma processing and fear extinction that may underlie symptomatic recovery from PTSD (Schnurr et al., 2022; Center for Substance Abuse Treatment (US), 2014). MDMA-Assisted Therapy may share this mechanism, given the association between MDMA administration and an increased capacity for all of these features (i.e., imaginal exposure, memory recall, and fear extinction; Maples-Keller et al., 2022; Young et al., 2015; Feduccia and Mithoefer, 2018). As such, the therapeutic framework used in the Phase 3 trials was well aligned with these aspects of evidence-based cognitive-behavioral approaches. Indeed, a number of cognitive processes and techniques were utilized (e.g., normalization, exposure, and cognitive reframing) in the service of an inner-directed, non-pathologizing approach to the participant’s experience. Therapists recontextualized the impact of trauma, supporting participants’ growing awareness of the ways in which their trauma history shaped their sense of self-worth, responsibility, shame, and related cognitions.

To complement the use of cognitive techniques, somatosensory trauma processing, which directly targets the physiological and sensory aspects of PTSD (Center for Substance Abuse Treatment (US), 2014; Grabbe and Miller-Karas, 2018), was also incorporated into the therapeutic approach. Somatic techniques (Weiss et al., 2015; Levine, 1997; Mindell, 2011; Levine, 2010) were used to increase the participant’s “window of tolerance” (i.e., their capacity for tolerating stressful experiences without entering a state of hyper-or hypoarousal; Siegel, 2020), expanding their ability to tolerate imaginal exposure and memory recall. As such, in its holistic approach, this therapeutic framework took advantage of complementary “top-down” and “bottom-up” processes that may underlie both the experience of PTSD and the therapeutic action of MDMA (Feduccia and Mithoefer, 2018; Wagner, 2021; Wagner et al., 2017; Avram et al., 2022; Borissova et al., 2021; Carhart-Harris et al., 2015; Gamma et al., 2000; Godes et al., 2023).

#### 2.3.1 Somatic experience and the non-pathologizing stance

In a biomedical model, certain somatic expressions of trauma are characterized as “symptoms” of PTSD—which is itself characterized as a “disorder”—and are therefore centered as pathological targets of treatment. By contrast, the organismic conceptual model used in the Phase 3 trials brought a non-pathologizing framework to such embodied responses as they emerged—often very intensely (Mithoefer, 2013)—within and outside the experimental sessions. This orientation—in which participants were invited to notice, without judgment, their embodied experience in the here-and-now (American Psychological Association, 2018)—required somatic awareness as well as intellectual and clinical humility on the part of each therapist, and was aligned with the many other schools of psychotherapy that aim to integrate an individual’s psychological (including cognitive and emotional), psychospiritual, and physical/somatic experience. These include, for example, the Gestalt (Perls et al., 1994), and Hakomi methods (Kurtz, 2007); process-oriented psychology (Mindell, 2011), internal family systems therapy (Schwartz, 1995; Falconer, 2023); transpersonal psychology (Grof and Valier, 1984; Nardini-Bubols et al., 2019), and a number of others.

The choice to take a non-pathologizing approach to the participant’s somatic experience was bolstered by prior MDMA research, in which the MDMA itself appeared to evoke strong physiological/somatic experiences in response to trauma-associated cognitive and emotional content that emerged during the session (Mithoefer, 2013; Greer and Tolbert, 1998; Greer and Tolbert, 1986; Passie, 2018). In many such cases, the MDMA experience was felt by participants to be more physically soothing than activating, thereby attenuating the aversive experience of trauma recall and processing (Wagner, 2021; Wagner et al., 2017)—i.e., increasing their “window of tolerance” (Siegel, 2020). However, in other cases (or even at different times during the same experimental session), the somatic expression of trauma was highly uncomfortable to the participant. Rooted in the concept of the inner healing intelligence, therapists working within this model of MDMA-Assisted Therapy took a non-pathologizing approach to *all* somatic expression, welcoming even uncomfortable emotional and physical experiences as valid and valuable forms of trauma processing. However, therapists were also attuned to where the participant was within their window of tolerance, and helped them utilize stress inoculation techniques and additional resources when the emotional and somatic experience of trauma processing risked becoming intolerable for the patient.

#### 2.3.2 MDMA and the “felt sense”

Operating within an organismic framework, psychotherapist and philosopher Gendlin (2007) used the term “felt sense” to describe an individual’s own intuitive, dynamic, and holistic internal experience as expressed in, or mediated by, interoception (Schmitt and Schoen, 2022). The hypoarousal and dissociation that may characterize a participant’s experience outside their window of tolerance represents and/or mediates a disconnection from this “felt sense,” which many (non-cognitive) trauma therapies therefore seek to restore (Perls et al., 1994; Weiss et al., 2015; Levine, 1997; Gendlin, 2007). During the Phase 2 trials, many participants reported that this felt sense emerged and/or was amplified in the MDMA-Assisted Therapy sessions (Godes et al., 2023). In some cases, this phenomenon was framed as a direct, unmediated experience of the concept of the inner healing intelligence, such that it was no longer merely a concept, but an intuitive, embodied experience of their own internal, self-directed recovery process (Godes et al., 2023). These felt experiences could often be recalled and evoked in the non-drug integration psychotherapy sessions, increasing the participant’s sense of safety and their capacity to engage with and process traumatic material.

#### 2.3.3 MDMA and emotional experience

In addition to focusing on a participant’s unfolding cognitive and somatic experiences, the therapeutic framework used in the Phase 3 trials also—and perhaps especially—emphasized a participant’s moment-to-moment emotional experience. Fear, anger, shame, and grief are among the many emotions that may emerge over the course of an intensive, trauma-focused therapy (Herman, 2015). Within the framework of MDMA-Assisted Therapy for PTSD, none of these emotions were pathologized or “explained away” (e.g., by attributing them exclusively to antecedent false beliefs, or by reducing them to mere activation of a dysregulated nervous system). Moreover, each participant set the pace for whether and how they noticed, “stayed with,” and/or deepened their emotional experience, with the therapist continually modeling trust in the participant’s inner healing intelligence. A non-pathologizing approach was brought even to intense emotional expression, consistent with a concept of organicity—and the attendant ethical stance—that welcomed all facets of human experience (Kohrt et al., 2020). Nevertheless, as noted above, therapists actively elicited discussions around the participant’s needs, and provided additional support as indicated.

## 3 Relationality

The concept of the inner healing intelligence described above does not imply that the therapeutic framework used in the Phase 3 trials of MDMA-Assisted Therapy for PTSD was individualistic. On the contrary, the concept of organicity situates an individual within complex, dynamic webs of interpersonal, transpersonal, communal, sociocultural, and environmental relationships (Weiss et al., 2015; Ogden, 2021). As such, like many therapeutic frameworks (Kurtz, 2007; Kuchuck, 2021; Ogden, 2015), the ethical stance of this model was fundamentally relational (Pollard, 2015): it conceptualized the therapeutic process as occurring in the relational field^11^ created by and between the participant, the two therapists, and the MDMA, and it considered how outside relationships (historical, familial, communal, social) influenced the participant’s experience of trauma and recovery in an ongoing way.

### 3.1 Autonomy and the “inner-directed” approach

Trauma expert Judith Herman writes (Herman, 2015) that the “first principle of recovery is the empowerment of the survivor” (p191). Within a trauma-informed conceptual framework (Box 4) that centers the participant’s access to their own inner healing intelligence, the efforts of a skillful therapist are directed toward amplifying the participant’s experience of empowerment and autonomy—of genuine choice, self-directed action, and self-efficacy. The term “autonomy,” as used here, is not to be understood as the participant’s ability to “manage” or otherwise *control* themselves or their environment. Indeed, individuals with a history of complex and persistent trauma may *suffer* their own extreme efforts to control their moment-to-moment internal experience (e.g., by avoiding certain places, even when the external circumstances no longer present a significant threat; Herman, 2015), generating a cognitive dissonance that represents a distressing experience of their own multiplicity. As noted above: within the organismic framework of MDMA-Assisted Therapy, therapists supported the participant by facilitating emotional and somatic processing without pathologizing any expressions of emotional/somatic intensity and/or multiplicity (Schwartz, 1995), and without rushing to “rescue” the participant (e.g., by immediately offering a grounding exercise during an apparent dissociative event). First and foremost, the therapists brought curiosity to the participant’s experience, and sought to clarify it—*without* asking the participant to change it. In so doing, the therapists modeled trust in the participant’s own inner-directed process, thereby fostering the participant’s empowerment and autonomy. In this way, participants cultivated greater awareness of their inner healing intelligence, the felt sense of which may strengthen their experience of trust in themselves, and their ability to embrace genuine autonomy (Rogers, 1965; Center for Substance Abuse Treatment (US), 2014; Herman, 2015).

Of note, certain structural aspects of this intervention may run counter to the participant’s experience of autonomy and empowerment, and therefore required explicit attention on the part of the therapists. For example, the co-therapy model (Box 5) affects the interpersonal dynamics at play in the therapeutic milieu, such that a participant may feel outnumbered by (presumably adversarial) “experts.” Another participant, perhaps especially one who has been deeply moved by the therapy experience (with or without MDMA), may struggle with termination-related feelings of separation, detachment, or abandonment, and/or fears of being “forgotten” by their study therapists. Such challenges are common in many relationally-oriented psychotherapies, but may be intensified by the fact that, in the model and protocol described here, the treatment relationship was brief—though the work done in that short time, and the bond created between the participant and their study therapists, may have been intense. Without careful attention, termination considerations, among others, might negatively affect the therapeutic relationship and/or undermine the therapists’ trauma-informed efforts to cultivate the participant’s sense of empowerment and self-directed change.

BOX 5 **The co-therapy model.**

A hallmark of this treatment is the co-therapy model, in which two therapists work together to support the participant’s inner-directed process. Such a model has been widely practiced in marriage and family therapy (Hendrix et al., 2001), and has been used in prior MDMA-Assisted Therapy with individuals, couples, and groups (Greer and Tolbert, 1998; Passie, 2018). The co-therapy model empowers the participant to autonomously engage each therapist in support of their own inner-directed process. As such, it may increase the depth and breadth of that process, e.g., by allowing the participant to explore multiple perspectives, and/or to notice how their experience (emotional, cognitive, somatic) is influenced by each therapist and/or the therapists’ interaction with one another. A full discussion of the co-therapy model is beyond the scope of this paper, but certain important features should be noted here. First, the cultural heritage and intersectional identities represented in the triad may be relevant to the therapeutic work, and may affect the participant’s willingness to disclose their prior trauma and/or their moment-to-moment experience within the therapeutic relationship (Norcross and Lambert, 2011). Second, unique transference-countertransference dynamics may also emerge in these interactions. To different degrees and in different ways, each therapist’s social identities and experiences may overlap with the participant’s—or a perpetrator’s, and/or that of another individual or group who has been a source of pain/trauma and/or safety, comfort, and protection. If skillfully navigated within the treatment (e.g., through the therapists’ collaborative exploration and mitigation of power imbalances), the resulting relational dynamics may offer opportunities for: (1) corrective interpersonal experiences, (2) a strengthened sense of psychological safety, and (3) a sense of personal empowerment that is at the core trauma-informed care generally, and this treatment model specifically. However, without careful attention on the part of the therapist, these dynamics are a potential source of interpersonal harm (West, 2013).

The MDMA-Assisted Therapy model described in this paper is intense for participants and therapists alike. All features described in the main text also apply to the co-therapy pair. For example, therapists respect one another’s autonomy; they strive to foster a sense of safety for one another; and they bring awareness to their and their co-therapists’ internal experience. In these and other ways, the co-therapists’ relationship with one another models the relationship and therapeutic approach used with the participant.

Each therapist’s self-awareness, self-care, and support system are essential for the ethical and sustainable practice of the therapeutic approach used in the Phase 3 trials of MDMA-Assisted Therapy for PTSD (Multidisciplinary Association for Psychedelic Studies (MAPS), 2022). The co-therapy model supports the therapists’ self-care (e.g., by allowing for breaks during long sessions), as well as the therapeutic process (inasmuch as each therapist brings different skills and relational dynamics to the relational field). The co-therapy model also facilitates each therapist’s professional development (e.g., taking time for debriefing, case consultation, and mutual support between sessions), and allows therapists to hold one another accountable for practicing with care and integrity, maintaining exquisite ethical and professional boundaries. All of these factors (personal, professional, ethical) are important in any kind of trauma-focused psychotherapy, but all the more so when working in an intensive model that includes lengthy experiences in non-ordinary states of consciousness (McNamee et al., 2023; Multidisciplinary Association for Psychedelic Studies (MAPS), 2022).

In any psychotherapy, the pace of change may shift in a moment-to-moment way, shaped in this case by a number of factors, including the shifting nature of the MDMA experience, as well as the participant’s sense of safety in the therapeutic milieu; manifestations of multiplicity; emotional and somatic experiences; sensitivity to and experience of each therapist; and access to a felt sense of their own inner healing intelligence. In practice, all of these experiences emerge within the relational field co-created in the triad, whereby each of the three contributes to and participates in verbal and nonverbal, conscious and unconscious, ways. As such, skillful therapists in the Phase 3 trials cultivated awareness of, and dynamically responded to, whatever was unfolding in that field, tracking the participant’s—*and their own*—internal (i.e., cognitive, emotional, interoceptive, somatic), interpersonal, and transpersonal experiences as they emerged in a moment-to-moment way (American Psychological Association, 2018).

### 3.2 Psychological safety and the therapeutic alliance

The primacy of the therapeutic alliance is a core tenet in many psychotherapy modalities (Frank et al., 1993; Norcross and Lambert, 2011; Norcross and Wampold, 2011; Wampold, 2015; Safran and Muran, 2003), and the relationship between the participant and the therapist may be particularly important in individuals with a history of complex trauma (Herman, 1998). In the relational framework of this therapeutic model, the participant’s recovery unfolds within a strong therapeutic relationship characterized by a sense of safety and trust. However, feelings of safety and trust may be hard-earned in participants with complex and persistent trauma: Judith Herman writes that although “the traumatized patient feels a desperate need to rely on the integrity and competence of the therapist, she cannot do so, for her capacity to trust has been damaged by the traumatic experience” (Herman, 2015; p. 198). In this model of MDMA-Assisted Therapy for PTSD, in a very short amount of time (i.e., three 90-min preparatory therapy sessions), therapists had to prove themselves to be trust*worthy*, bringing bring care, compassion, attunement, and patience to the therapeutic relationship, and surrendering their own timeline or agenda, knowing that it was *their own* responsibility to create and maintain a space in which the participant feels safe, rather than it being the participant’s responsibility to trust the therapist(s; Redpath, 2019; Herman, 1998). Establishing and maintaining this sense of psychological safety and trust is paramount to a participant’s trauma processing and growth.

This sense of safety does not equate with invulnerability. On the contrary: vulnerability—defined by Brown (2015) as a feeling of “uncertainty, risk, and emotional exposure” that is accompanied by a felt experience of *instability*—is an important element of trauma recovery, and emerges as a trusting therapeutic alliance is established. Expansion of a patient’s window of tolerance may require that the participant first venture into experiences that are highly aversive, as seen, for example, in Prolonged Exposure therapy for PTSD. In the model described here, the two therapists were responsible for creating a therapeutic container in which the participant felt safe *enough* to be vulnerable, while making it clear that they respected and deferred to the participant’s choice regarding if and when they chose to engage in trauma processing. This therapeutic approach invited and welcomed the participant’s vulnerability—including, not despite, the attendant experience of instability. Crucially, the therapists led with their *own* vulnerability—modeling their stated values in their willingness to be present with their own uncertainty and exposure, as these emerged in the therapeutic act of witnessing (Blackwell, 1997; Goodman, 2012). The participant was thereby encouraged, implicitly and explicitly, to explore their capacity to trust both their own inner healing intelligence and the therapists.

To establish and maintain a therapeutic container that is worthy of the participant’s trust, MDMA therapists must be scrupulous in their attention to professional boundaries. This is true in any psychotherapy; however, it may be particularly important when working with participants in non-ordinary states of consciousness (Brennan et al., 2021). MDMA increases a participant’s sense of social intimacy (Wagner et al., 2021), their perception of others’ trustworthiness, and their tendency to cooperate (Borissova et al., 2021; Gabay et al., 2019; Stewart et al., 2014). These effects may be relevant to the therapeutic value of the experimental sessions in MDMA-Assisted Therapy; however, they may also amplify power differentials, which are inherent in any clinical relationship (Brennan et al., 2021; Reandeau and Wampold, 1991). As such, therapists on the Phase 3 studies were expected to adhere to the highest standards of personal and professional integrity and ethical conduct, as laid out in the MAPS Code of Ethics (Multidisciplinary Association for Psychedelic Studies (MAPS), 2022), and as confirmed by high fidelity to the model, evaluated through clinical consultation and video review by independent adherence raters.

### 3.3 Psychodynamic awareness

Psychodynamic awareness (i.e., an awareness of the conscious and unconscious factors—static and dynamic, implicit and explicit, verbal and non-verbal—at work in the treatment) is required for a therapist seeking to establish a working alliance within which the participant and the therapist both feel safe enough to explore their internal experience and take risks (e.g., by confronting difficult emotions, challenging ingrained beliefs), noticing and responding to their experience of one another in the relational field (Kuchuck, 2021; Gelso and Carter, 1994). Psychodynamic awareness is therefore essential for any kind of intensive trauma therapy. For this reason, in MDMA-Assisted Therapy, the therapists strived to be attuned to the participant’s, their co-therapist’s, and their own internal experience, knowing that clarity about one requires and facilitated clarity about the others (Geller et al., 2010; Gelso and Carter, 1994).

The psychodynamic phenomena of transference and countertransference have been described in many ways, but in this case will be used to refer to the full range of (often initially unconscious) responses the participants and therapists experience towards one another, informed by their individual developmental histories, as well as the real relationship that is unfolding in the here-and-now. In the context of MDMA-Assisted Therapy, the Phase 3 therapists worked to notice the ways in which these phenomena were manifesting in the relational field in a moment-to-moment way. For example, one therapist’s physical appearance may have reminded a participant of their perpetrator, precipitating an intense emotional and/or somatic expression from the participant. This intense expression could be highly activating for the second therapist, who may have defended against feelings of helplessness and emotional overwhelm by withdrawing relationally, or by making a grandiose and intrusive attempt to “rescue” the participant (or the first therapist; Herman, 2015). Although not uncommon, neither of these reactions would be consistent with a conceptual framework intended to foster a sense of safety, bringing a non-pathologizing stance to intense expression and amplifying the participant’s experience of empowerment and autonomy. Indeed, left unconscious, countertransference may represent “the single most important obstacle to successful psychotherapy” (Strupp, 1980)—particularly a therapy built on a conceptual framework that centers the participant’s inner healing intelligence as the agent of change. The extended duration of the therapy sessions in this model—including the 8-h experimental sessions and the 90-min non-drug psychotherapy sessions alike—may have amplified these relational dynamics and provided more opportunities for deeper content to emerge and shift in the relational field. This has been seen in the (non-psychedelic) psychoanalytic work of Bollas and Bollas (2013), and may be further enhanced by MDMA. The therapists therefore aimed to bring such intra-and interpersonal dynamics into their own conscious awareness, without pathologizing them. Indeed, to pathologize them might have distanced them from the participant and/or themselves, obscuring the complexity at work within and between the three members of the therapeutic triad, and implicitly undermining the stated values of the psychotherapy framework. Moreover, “effective *use* of countertransference reactions may be of profound benefit to the therapy” (Gelso and Carter, 1994; italics added), because the therapist’s conscious awareness of their own internal and behavioral responses may help to clarify their understanding of the participant, themselves, and the process that is unfolding in the relational field (Kuchuck, 2021). For these reasons, therapists were instructed to bring non-defensive curiosity to the dynamic factors at work in that field, and invited the participant to do the same, allowing their shared discoveries to inform what followed in the treatment.

Sensitivity to a therapists’ verbal and nonverbal cues, and their own prior and ongoing experiences of shame, guilt, and estrangement, as well as their struggles with trust, attachment, self-compassion, and forgiveness—all of these are common in individuals with a history of complex and persistent trauma (Herman, 2015), and may affect the strength and perceived safety of the therapeutic milieu. Each therapist likewise brings their own history, sensitivity, vulnerability, self-awareness, and therapeutic presence to their work, and may not be empathically attuned to the participant at all times (Kuchuck, 2021; Geller et al., 2010). Empathic ruptures are therefore inevitable in any intensive relational work. In the context of MDMA-Assisted Therapy, the intrapersonal, interpersonal, and other contextual factors that may contribute to these ruptures were noticed by skillful therapists; however, therapists were instructed not to pathologize them and/or explicitly seek to avoid them. Indeed, repeated cycles of rupture and repair may actually facilitate the corrective emotional and attachment experiences that are required to move towards healing from interpersonal trauma (McLaughlin et al., 2014). The increased sensitivity, empathy, and interpersonal closeness induced by MDMA, coupled with the length of the sessions, may facilitate such cycles.^12^

All of the considerations above apply to non-drug psychotherapy and experimental therapy sessions alike. However, in experimental sessions specifically, MDMA itself also likely contributed to the relational field, as its pharmacological effects can facilitate or amplify many of the aforementioned factors (e.g., the experience of psychological safety and interpersonal closeness, the emergence of charged affective and/or somatic experiences, the repair of empathic ruptures that are essential for trauma processing; Herman, 2015; McLaughlin et al., 2014). MDMA may also be a target of the participant’s transference, inasmuch as their prior experiences (e.g., exposure to positive or negative media coverage of MDMA, prior use of MDMA) may shape their hopes, fears, and expectations before and after the three experimental sessions. A full description of how MDMA may directly affect transference reactions is outside the scope of this paper, but these psychodynamic considerations bear future study.^13^

### 3.4 Sociocultural humility

Conventional psychotherapy models developed within a Western, White/White American, often male, middle class, heterosexual, cisgender framework have significant limitations, particularly for addressing the concerns of individuals from diverse sociocultural backgrounds (American Psychological Association, 2017; American Psychological Association, 2003). All interactions between participants and therapists occur within a cultural context, so a trauma-informed therapist seeking to (1) establish a participant’s sense of safety and self-empowerment, and (2) foster the participant’s self-directed healing process, must bring self-awareness and sociocultural humility to their work. Here we use the term “sociocultural” broadly, to include many different aspects of a participant’s identity (including, but not limited to, their cultural heritage, race, ethnicity, socioeconomic circumstances, gender, sexuality, experiences of marginalization/oppression). By “humility” we mean that the therapist brings “beginner’s mind” to the question of sociocultural identity. Rather than assuming that differences do not exist and/or are irrelevant to the treatment, therapists were instructed to openly and actively invite the participant to explore with them the ways in which sociocultural similarities and differences are at work in the therapeutic relationship (American Psychological Association, 2017). Outside the sessions, the therapist’s own non-defensive self-examination (e.g., through personal therapy, professional consultation) was and is critical for developing cultural humility, which in turn is important for building a strong therapeutic alliance, navigating differences in cultural experiences and values, and recognizing and repairing culture-related ruptures (Mosher et al., 2017). As Mosher and colleagues note (Mosher et al., 2017): “When engaging with clients, it is important to recognize our limitations, remain self-aware, and remember that *the client is the expert* on their unique set of cultural identities and experiences. The culturally humble therapist engages with the client in a way that *co-creates a relational experience*. In doing so, the *connection between clients’ and therapists’ cultural values and beliefs are part of the fuel for a deeper relational connection* …. Throughout the process, therapists should be *aware of their positionality in the relationship*, *and while being genuine and real, should take steps to mitigate their power and influence*” (Mosher et al., 2017; p. 226; italics ours). Cultural humility is therefore essential to the inner-directed, relationally-oriented, and trauma-informed framework of MDMA-Assisted Therapy.

Within that framework, therapists on the Phase 3 trials sought to bring curiosity and awareness to the implicit and explicit dimensions of identity and social location (Lee et al., 2022) represented in the triad, any of which might affect the therapeutic alliance and the transference-countertransference dynamics at work in the relational field. Without each therapist’s explicit attention, their own intersectional identities and social location might engender and/or recapitulate harmful relational dynamics (e.g., dependency, implicit or explicit power struggles) in the participant’s life, impeding efforts to foster the participant’s trust and autonomy (Reandeau and Wampold, 1991; Gelso and Carter, 1994). For this reason, in MDMA-Assisted Therapy, therapists aimed to (1) bring attention to these various dimensions, demonstrating their own ability to notice and name the many factors that may shape and distort the relational field, and (2) make a conscious effort to mitigate any imbalances of power that might emerge within it.

### 3.5 Healing in community

In the model described here, the practice of MDMA-Assisted Therapy itself is triadic, unfolding in the intersubjective space between the participant and the two therapists. However, an organismic, trauma-informed framework also highlights the importance of “matrix” (Box 2), i.e., the factors *outside* the therapeutic container that nevertheless can influence trauma processing and symptomatic improvement (Eisner, 1997). These may include interpersonal (e.g., family) relationships*,^14^ negative exchanges with others (e.g., social ostracism vs. social acknowledgement; racial/ethnic and gender−/sexuality-based micro-and macroaggressions), and broader sociocultural factors (e.g., individualistic vs. collectivistic approaches to trauma and recovery) that contribute to the course and severity of PTSD (Herman, 2015; Herman, 1998; Maercker and Horn, 2013; Herman, 2023).

Indeed, one challenge in this therapeutic model is the fact that the therapeutic container, even skillfully maintained, cannot compensate for the dominant cultural framework of psychological healing in the Eurocentric (Bhambra and Holmwood, 2021; Andrews and Sutphen, 2003) biomedical model, which is marked by a prominent lack of community-based support for collective healing and growth (Josewski et al., 2023), and by a tendency to pathologize the individual, thereby obscuring social conditions that themselves may be inherently traumatic and in need of change (Rosenthal et al., 2016). Herman (1998) notes that “Trauma destroys the social systems of care, protection, and meaning that support human life. The recovery process requires the reconstruction of these systems.” No matter how strong the therapeutic relationship, that “reconstruction” cannot take place within an individualistic model of trauma and recovery. Such a model stands in stark contrast to the community-based approaches that are characteristic of Indigenous practices with psychoactive substances (Ona et al., 2022). Pragmatically, collaborative decision-making for continued psychosocial support (e.g., clinical, community, family, peers) is often indicated to support the therapeutic process as it continues to unfold upon termination from the study. However, future research will be required to identify the ways in which communal and cultural dimensions support (and/or hinder) individual and collective healing.

## 4 Discussion

This paper describes many of the historical and contemporary therapeutic principles that contributed to the conceptual framework of MDMA-Assisted Therapy for PTSD used in the Phase 3 trials. This therapy model may be particularly useful when working with MDMA; however, within-group analyses in the Phase 3 trials identified a significant treatment effect of the psychotherapy alone, even in participants presenting with severe and persistent symptoms despite previous evidence-based interventions. These findings lend empirical support to the therapy model, even without the addition of MDMA. As such, a fuller exploration of the core concepts that underlie the therapeutic approach is warranted. While the various concepts, theories, and practices informing the approach to MDMA-Assisted Therapy are not original, their synthesis into an integrative—rather than a simply eclectic (Arkowitz, 1989)—approach is novel. This paper addresses a key gap in the literature by articulating the conceptual framework for the therapeutic approach, and by considering the specific implications of its use in conjunction with MDMA.

In describing the conceptual framework, various individuals and multiple “schools” of psychotherapy have been referenced above. Many of these directly and indirectly shaped the investigators who developed the model (Box 3), and many aspects of other therapeutic modalities are also in alignment with different features of this conceptual framework. However, this model does not comprise a random collection of miscellaneous principles. On the contrary, it is an internally coherent, trauma-informed conceptual framework—centering organicity and the attendant concepts of the inner healing intelligence and relationality—in which therapists take an active role by fostering the interpersonal and environmental conditions needed to support the participant’s agency and autonomy in their process of self-directed healing and growth.

For clinicians working with MDMA-Assisted Therapy for PTSD, familiarity with these core concepts is important, as they directly informed the therapeutic approach that led to the impressive clinical outcomes on the Phase 3 trials. However, by naming a small group of clinicians and theoreticians whose work strongly influenced this therapeutic model, this paper may give the false impression of a direct theoretical lineage, idealizing and/or exaggerating the influence of a small number of individuals (generally from dominant social identities, within a largely individualistic biomedical model that centers physicians over the many other caregivers that sustain a community; Andrews and Sutphen, 2003; Josewski et al., 2023) at the expense of the many other influences that shaped the therapeutic framework and approach. As noted in Box 3, those influences were no less essential, and included not only the many others who have advanced—often without credit—psychedelic and psychotherapy research and practice, but the many intrapersonal, interpersonal, cultural, and social factors that shaped the investigators who developed the therapy model.

This unique psychotherapy platform—which was manualized but unscripted—cannot be fully captured and/or conveyed didactically. On the contrary, the treatment is experiential for participant and therapist alike. Although the participant’s inner healing intelligence is the primary agent of change, that change occurs within the context of a strong therapeutic relationship, in which the therapist tends to the many verbal and nonverbal factors that facilitate or impede the participant’s sense of psychological safety and trust, and the therapist’s willingness to exercise humility in the therapeutic milieu. As such, although the participant is the “expert” in their own healing process (Kohrt et al., 2020), therapists must have expertise in order to supply the facilitative conditions for that process in a way that is consistent with the core conceptual framework described here. To be sure, the efficacy and safety data reported in the clinical trials of MDMA-Assisted Therapy for PTSD was generated by therapists who participated in an extensive training program that extended beyond the therapy manual, and continued to refine their practice in close consultation with a master clinician and adherence raters who observed their video-recorded sessions. To further their education experientially, most therapists in the Phase 3 trial also had the voluntary opportunity to participate in a clinical trial (NCT01404754) in which they could receive MDMA in a therapeutic setting, supported by a master clinician. Participation in this experiential component was not required; nor have its effects on therapeutic efficacy been studied to date, though this is an important area of future research (Wilson-Poe et al., 2024). What is clear is that, given the complexity of the theoretical framework and the nuances of the therapeutic approach, should investigational MDMA-Assisted Therapy eventually receive FDA approval for use in the treatment of individuals with PTSD, it will likely require administration by skilled therapists with specialized training and consultation.

The use of MDMA within more conventional therapeutic models is another area of active investigation (Wagner et al., 2019). Indeed, because MDMA may directly affect certain biological mechanisms of post-traumatic memory processing (Feduccia and Mithoefer, 2018), and because of its effects on a participant’s experience of interpersonal closeness (Borissova et al., 2021; Kirkpatrick et al., 2014; Hysek et al., 2014), it may be a useful adjunct to a number of trauma-centered and relational psychotherapies. Nevertheless, the conceptual framework articulated here stands in contrast to a pathology-based biomedical model that favors diagnoses and labels (e.g., “treatment-resistant,” “personality-disordered”) that may reduce the participant’s lived experience to a constellation of internal and external “symptoms” representing an internal deficit that requires external, “expert” intervention. Such an individualistic, deficit-based model is inconsistent with the concept of organicity, in which the participant is 1 whole (i.e., neither “broken” nor otherwise defective), and 2 in a dynamic, bidirectional relationship with their external environment. Although clinical outcomes in the Phase 3 trials were quantified in terms of a change in symptom severity, the therapeutic framework conceptualized “healing” more broadly (Kohrt et al., 2020), in which the participant, rather than the therapists, guided the pace and direction of change.

Papers reporting primary outcomes in psychedelic clinical trials do not usually include an in-depth discussion of the psychotherapy platform, so they may lead the reader to underestimate the extent and importance of the therapy. This paper aims to begin to correct that misunderstanding, and a subsequent article on therapeutic process will be important to continue that effort. Moreover, additional psychotherapy research should interrogate the relationship between therapists’ theoretical frameworks, the resulting behaviors of therapists and patients, and clinical outcomes. Indeed, the efficacy of the therapeutic approach may have little do to with the theoretical framework described above, and may instead (or additionally) represent “general change mechanisms” (Wolff et al., 2024) or “common factors” (Frank et al., 1993) at work across therapeutic modalities. This is an important area for future psychotherapy process research, which investigates not clinical outcomes (as measured, for example, in comparative efficacy studies), but the therapeutic processes (e.g., therapeutic priming; facilitation of emotional breakthroughs; activation of internal and external resources) that may underlie long-term change (Wolff et al., 2024).

## 5 Conclusions and future directions

The therapy model used in the Phase 3 trials of MDMA-Assisted Therapy for PTSD was shaped by a coherent conceptual framework that centered the participant’s self-directed journey toward healing and growth (Kohrt et al., 2020), facilitated by highly trained, empathically attuned, relationally skillful, trauma-informed therapists. This complex model warrants further study, particularly because within-group analyses of the Phase 3 trials identified a significant within-group effect in the placebo + psychotherapy group—though it is unclear to what extent participants’ and/or therapists’ expectations contributed to these outcomes (Muthukumaraswamy et al., 2021). Head-to-head comparisons of psychotherapy platforms will be important areas of future research, and must control for the extended duration of contact in the present model. Further research should also investigate whether MDMA amplifies the therapeutic utility and/or retention rates for various evidence-based treatments for PTSD. An additional area of study will be the optimization of the therapeutic effect of MDMA (e.g., whether the effect size is larger when MDMA is administered in a group setting, combined with current evidence-based therapies, or other psychological support models; Anderson et al., 2020; Kettner et al., 2021; Passie, 2012). As noted in the foregoing paragraph, in addition to such outcomes research (i.e., comparative efficacy trials) and mechanistic studies, psychotherapy *process* research—which investigates “what takes place between, and within, the patient and therapist during the course of their interaction” (Ardito and Rabellino, 2011)—should be undertaken to identify the common and specific factors underlying the clinical benefits of the therapeutic approach used in the Phase 3 trials for moderate and severe PTSD (Wolff et al., 2024). As MDMA-Assisted Therapy research continues to evolve, an awareness of its conceptual roots may be essential for honoring the complexity of a participant’s journey toward healing and recovery.

## Data Availability

The original contributions presented in the study are included in the article/supplementary material, further inquiries can be directed to the corresponding author.

## Footnotes

^1^Note that the therapeutic approach was never intended to be a standalone treatment for PTSD, making the findings in the placebo + psychotherapy even more remarkable.

^2^The authors thank Trent S. Riney for this apt metaphor.

^3^As noted, the concept of organicity is also implicit in certain Indigenous, Eastern, and Western esoteric traditions (von Goethe and Miller, 2009; Harrington, 1996; Redpath, 2019), some of which informed the conceptual framework described above (Box 3). As such, the idea long predates Goldstein’s seminal work. However, his articulation of the concept was particularly striking at the time, as it stood in opposition to the deficit-based, deterministic models of early psychoanalysis and behaviorism. It also directly influenced the founders of humanistic psychology and formed the starting point of empirical psychological research into what the field’s founders called a person’s “actualizing tendency” and the “facilitative conditions” provided by the therapists (Maslow, 1968; Rogers, 1965; Frick et al., 1971). Together, these two concepts represent the two pillars described throughout this text as the “inner healing intelligence” and “relationality,” respectively. Given the relevance of Carl Rogers’s and others’ work (particularly regarding the “facilitative conditions”) to contemporary psychotherapy more generally, we expect that much of the discussion of relationality below will be familiar to the reader who is a trained psychotherapist, given the integration of Rogerian concepts into contemporary teaching and practice.

^4^Inner healing intelligence” is essentially a technical term, as discussed in the main text. Outside (though related to) this concept—and as used in this paper—the term “healing” (or “recovery”) more generally refers to a self-directed process by which an individual comes to flourish in and despite adversity, increasing their own understanding of who they are as individuals—separate from any developmentally-and trauma-based conditioning—thereby strengthening trust in their own capacity to see, care for, and integrate aspects of themselves, all while recognizing their connection to a larger community (Kohrt et al., 2020). The process of healing, thus defined, often brings forth a rise in self-confidence, an updated personal narrative, and improved relationships, as well as a greater ability to tolerate uncertainty, ambiguity, and paradoxicality. Such changes may or may not be measurable by standard assessments of PTSD symptomatology; however, Phase 2 and Phase 3 trials indicate that this process reduces the presence and severity of distress attached to prior narratives and memories, as evidenced by CAPS scores and measures of disability (Mitchell et al., 2021; Mitchell et al., 2023).

^5^Note that the inner healing intelligence, as used here, is not another “organ” within the “organismic” framework discussed in this paper. The inner healing intelligence is a *capacity* and a *process*; crucially, it is not a material entity. (The term “inner healer” is an imperfect substitute for “inner healing intelligence,” because “healer” may connote a concrete, independent being—internal and/or spiritual—in which a participant has to “believe” in order to engage meaningfully in this therapeutic modality.) Despite the possibility of confusion, we use the term “inner healing intelligence” because of its historical meaning and theoretical lineage; however, some readers may nevertheless find it alienating. In such a case, substituting “the organism’s natural capacity to heal, given the facilitative conditions” should suffice for clarity. Of note, like the reader, patients were not required to adopt the term “inner healing intelligence” during these trials. The concept was introduced early in the preparation phase, consistent with Frank’s “common factors” model of psychotherapy, in which the therapist “acculturates” the patient to the treatment model, providing an account by which the treatment may facilitate healing. However, in an effort to (1) maximize the participant’s autonomy, (2) embody cultural humility, and (3) minimize the inherent power imbalance in the therapeutic relationship, therapists were trained to explore and use concepts and terminology that resonated with each patient’s unique experience, culture, and worldview. Importantly, this meant that the term “inner healing intelligence” was not used at all in the therapeutic process for some participants.

^6^More recently, models of cognitive behavioral therapy, while quite directive in content and technique, have nevertheless implicitly incorporated the concept of the inner healing intelligence by formulating PTSD as an interruption of the natural recovery process (Wagner et al., 2019). The concept is also becoming a subject of research in its own right (Peill et al., 2024).

^7^This paper describes the conceptual framework for MDMA-Assisted Therapy for PTSD, an investigational treatment that has only been systematically studied within the context of a research protocol. As such, the therapists on these trials were clinical investigators. Nevertheless, the conceptual framework described here preceded—and informed—the therapeutic approach that was studied in the Phase 3 trials. As such—and in alignment with the psychotherapy literature—we use the term “therapists,” rather than “investigators,” in this paper to refer to the core group of clinician-researchers who developed this therapeutic approach, and to the investigators who were trained and/or supervised by them on the Phase 3 trials.

^8^In early versions of the Phase 3 MDMA psychotherapy manual, the term “non-directive” was used to describe this Rogerian aspect of the therapeutic approach. However, the term was later changed to “inner-directed,” because of confusion that often emerged when the term “non-directive” was understood colloquially (Horton et al., 2021; Deckel et al., 2024). This colloquial reading led Fischman (2019), for example, to describe the therapeutic approach to MDMA-Assisted Therapy as giving “generic advice to follow the patient’s’inner healing intelligence’” (p. 72), at the expense of deeper exploration of the content and meaning of the unfolding experience. As noted in the main text, this characterization does not capture the nuanced therapeutic approach adopted in the Phase 3 trials.

^9^Please note that the overlap between these concepts should not be taken to mean that the theoretical framework adopted wholesale the ideas promoted by the authors in question. The same is true of the influences noted elsewhere in the paper, including the main text, footnotes, and inset boxes.

^10^Indeed, the absence of a role for individual self-actualization and psychospiritual experience within these frameworks was what prompted the development of humanistic psychology (Bland and DeRobertis, 2020), from which transpersonal psychology later emerged (Frick et al., 1971). However, the fields of psychoanalysis and behavioral psychology have expanded and matured extensively since that time, and in some cases now include transpersonal elements, as seen in the psychoanalytic work of Eigen (1998) and Pearson and Marlo (2021), and in the incorporation of Eastern contemplative practices into third-wave cognitive behavioral therapies (Dimidjian and Linehan, 2003; Kabat-Zinn, 2003). Given this rapprochement, many of the concepts throughout this paper will be familiar even to therapists who lack experience or association with humanistic/transpersonal schools. That said, unlike the third-wave cognitive behavioral therapies, the conceptual framework described in this paper leaves space for ontological uncertainty (i.e., mystery) around transpersonal and other experiences, which is rare in modern evidence-based psychotherapies, but increasingly understood to be an important dimension of intellectual and clinical humility (Michalec et al., 2024; Bąk et al., 2022), both of which are crucial components of the ethical stance of this therapeutic framework. Just as this model does not pathologize the participant’s experience, nor does it entirely “psychologize” that experience by filtering it through the highly individualistic lens of most evidence-based interventions. Consistent with the modern psychological concept of “therapeutic presence” (Geller et al., 2010), the “loving presence” in Hakomi (Weiss et al., 2015), and the Buddhist concept of “beginner’s mind” (Suzuki, 2006)—which has been adopted, albeit in an entirely secularized form, by modern, third-wave cognitive behavioral therapy (Dimidjian and Linehan, 2003; Kabat-Zinn, 2003)—therapists in the Phase 3 trials brought mindful, compassionate, and non-judgmental curiosity to the participant’s experience in the here-and-now, welcoming uncertainty and mystery, while inviting participants to do the same.

^11^The concept of the relational (or intersubjective) field is well articulated in the interpersonal/relational psychoanalytic tradition (Kuchuck, 2021; Ogden, 2004), some aspects of which are represented in MDMA-Assisted Therapy. Psychoanalyst Stern (2017) notes that the client (in this case, the participant) and the therapist(s) each participate in the treatment: they “are continuously and inevitably, and consciously and unconsciously, in interaction with one another. This interaction has to do with what they experience in one another’s presence, and how they behave. The field also determines what each participant can experience in the presence of the other, especially the affective aspects of experience. The field is, on one hand, the sum total of all those influences, conscious and unconscious, that each of the analytic participants exerts on the other. On the other hand, the field is the outcome of all those influences, the relatedness and experience that are created between the two people as a result of the way they deal with one another.” Understood in this psychodynamically-informed way, the relational fabric of MDMA-Assisted Therapy extends beyond a straightforward concept of the therapeutic alliance, favoring the complex relational field as the vehicle for therapeutic processing and change (Kuchuck, 2021).

^12^In some cases, a rupture to the therapeutic alliance may emerge after even a “positive” experience in the experimental session. For example, a participant who unexpectedly gave themselves permission to be vulnerable during the session, bypassing standard modes of self-protection, might later experience a “rebound” destabilization. Such a response is neither rare nor pathological; therapists were instructed to normalize it, recalling the non-linear nature of trauma processing and recovery, while also providing additional support as needed.

^13^We thank Dr. Harvey Schwartz (*personal communication*) for his insights into how the experimental medicine itself is a transference object, and we look forward to future scholarship on the topic.

^14^The Phase 2 and 3 studies were conducted in North America, Europe and Israel—cultures in which most people lacked understanding of the nature or healing potential of NOSC, and were likely to judge or dismiss an MDMA experience as mere intoxication. Therefore, an important aspect of preparation and integration was to prepare each participant to be discerning about how or with whom they shared their experiences with individuals outside the study.
